# Supplementary material for: TENT2, TUT4, and TUT7 selectively regulate miRNA sequence and abundance
Source: Nat Commun. 2022 Sep 7;13:5260. doi: 10.1038/s41467-022-32969-8 (PMC9452540; doi:10.1038/s41467-022-32969-8)
Supplement: Supplementary file 2 — Supplementary Information [file 41467_2022_32969_MOESM2_ESM.pdf]

Supplementary Information

TENT2, TUT4, and TUT7 selectively regulate miRNA sequence and abundance

Acong Yang[1,#,] Xavier Bofill-De Ros[1,#,] Ryan Stanton[1], Tie-Juan Shao[1,2], Patricia Villanueva[1], Shuo Gu[1*]
[1]RNA Mediated Gene Regulation Section; RNA Biology Laboratory, Center for Cancer Research, National Cancer Institute; Frederick, MD, 21702; United States.
[2]School of Basic Medicine, Zhejiang Chinese Medical University; Hangzhou, 310053; China

[#] These authors contributed equally
[*] Correspondence to: shuo.gu@nih.gov

Supplementary Figures 1-7
Supplementary Tables 1-2

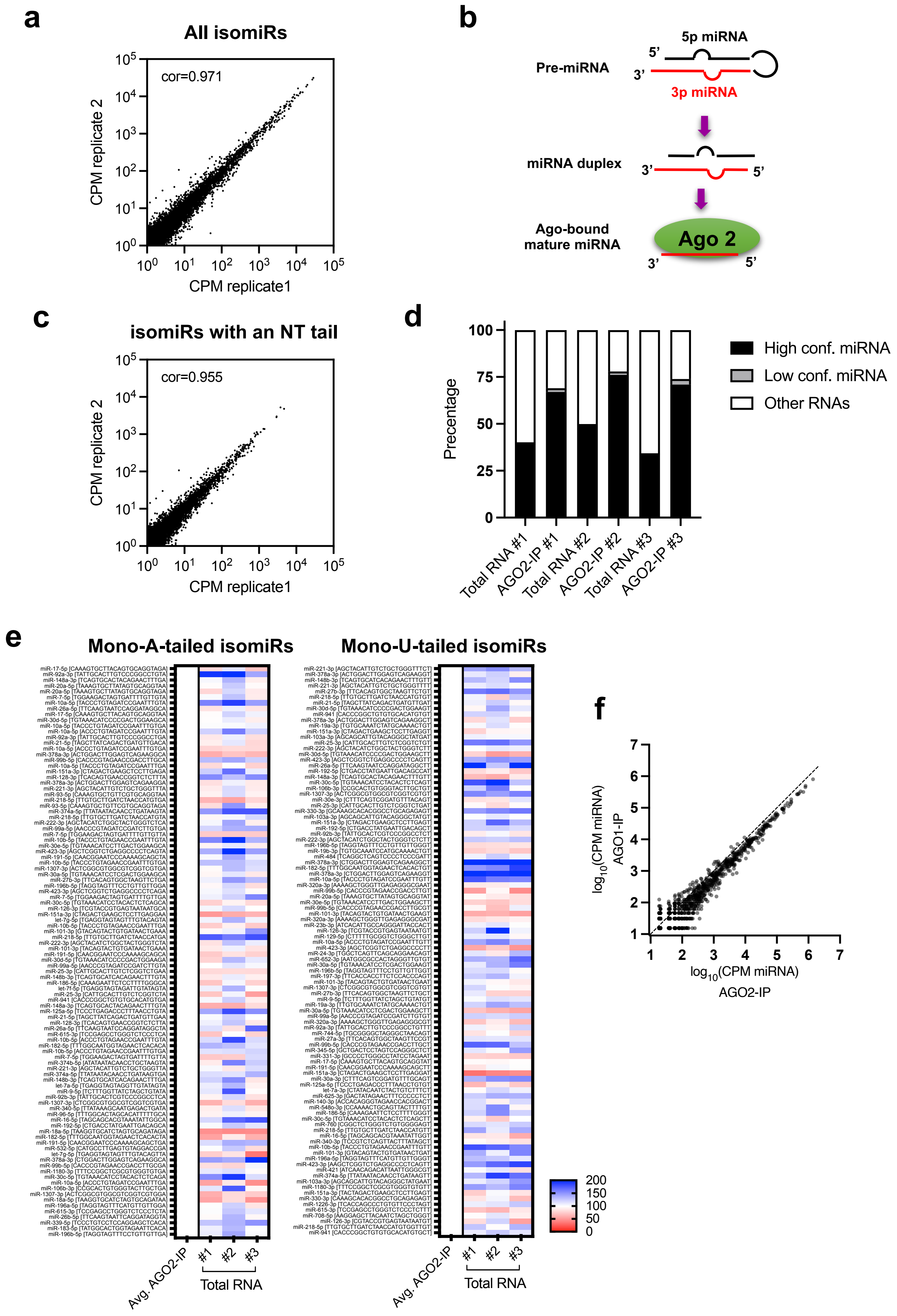
a
All isomiRs
cor=0.971
CPM replicate 2
CPM replicate1
b
Pre-miRNA
5p miRNA
3p miRNA
5'
3'
miRNA duplex
3'
5'
Ago-bound
mature miRNA
Ago 2
3'
5'
c
isomiRs with an NT tail
cor=0.955
CPM replicate 2
CPM replicate1
d
Precentage
100
75
50
25
0
Total RNA #1
AGO2-IP #1
Total RNA #2
AGO2-IP #2
Total RNA #3
AGO2-IP #3
High conf. miRNA
Low conf. miRNA
Other RNAs
e
Mono-A-tailed isomiRs
Mono-U-tailed isomiRs
Avg. AGO2-IP
#1
#2
#3
Total RNA
200
150
100
50
0
f
log10(CPM miRNA)
AGO1-IP
log10(CPM miRNA)
AGO2-IP

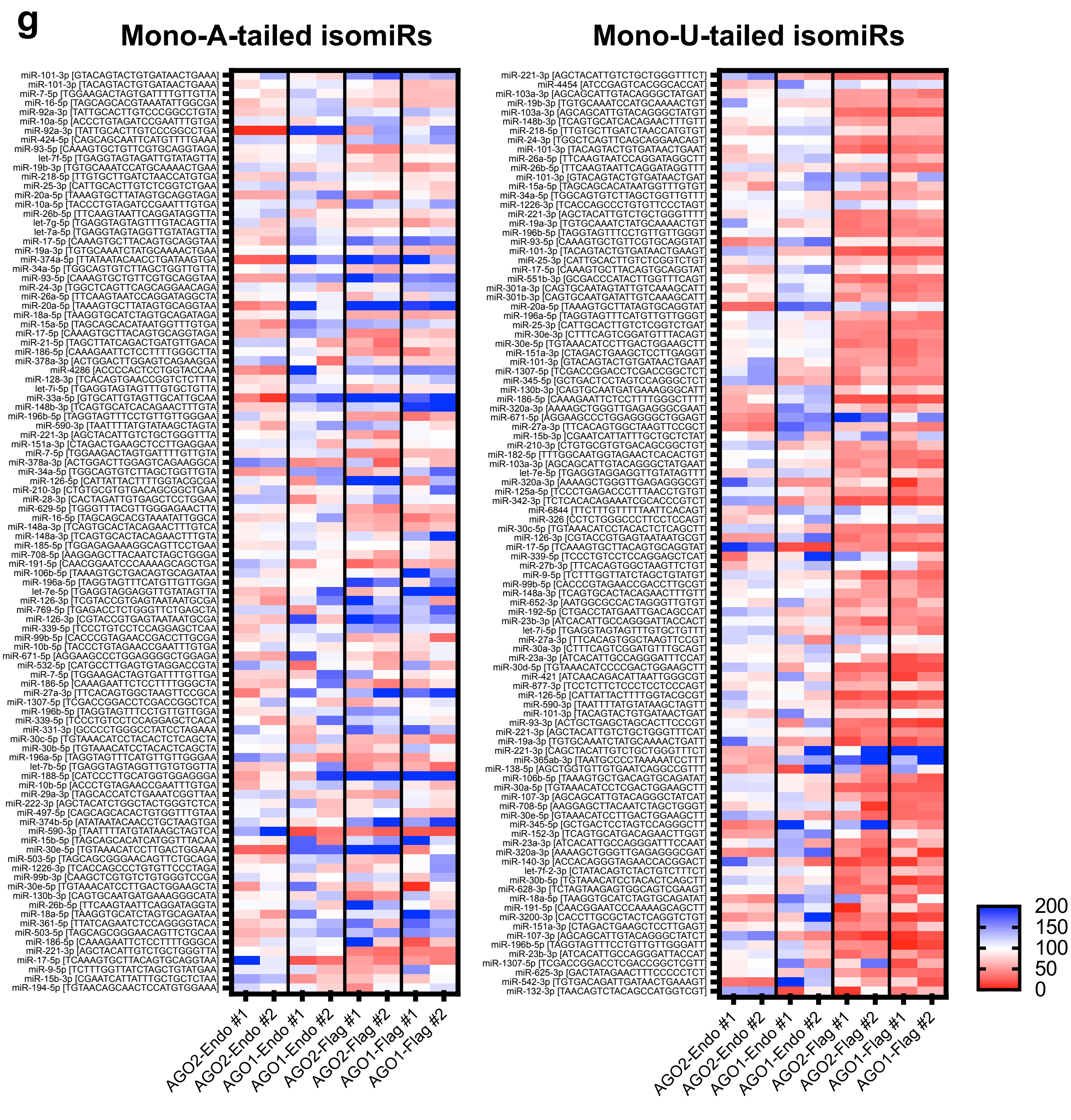

Supplementary Fig. 1 miRNA 3' tailing is frequent and specific.

a, c Scatter plots of isomiR abundance (counts per million, CPM) among the two replicates of HEK293T (WT). Correlations (cor) were calculated using the Pearson coefficient. **b** Scheme of miRNA biogenesis. Note that the 3' end of the 3p miRNA is the same as that of the pre-miRNA while the 3' end of the 5p miRNA is not accessible at the pre-miRNA stage. **d** Analysis of the composition from small-RNA-seq of total and IP samples. **e** Heatmaps of the relative abundance of mono-tailed isomiRs (sequences are indicated in square brackets) between AGO2-IP and Total RNA samples. Colorimetric scale ranges in values between 0 and ≥200. **f** Scatter plot of miRNA abundances between total AGO1-IP and AGO2-IP. **g** Heatmaps of the relative abundance of mono-tailed isomiRs (sequences are indicated in square brackets) between endogenous and ectopic expressed AGO1 and AGO2 pull-downs. Colorimetric scale ranges in values between 0 and ≥200.

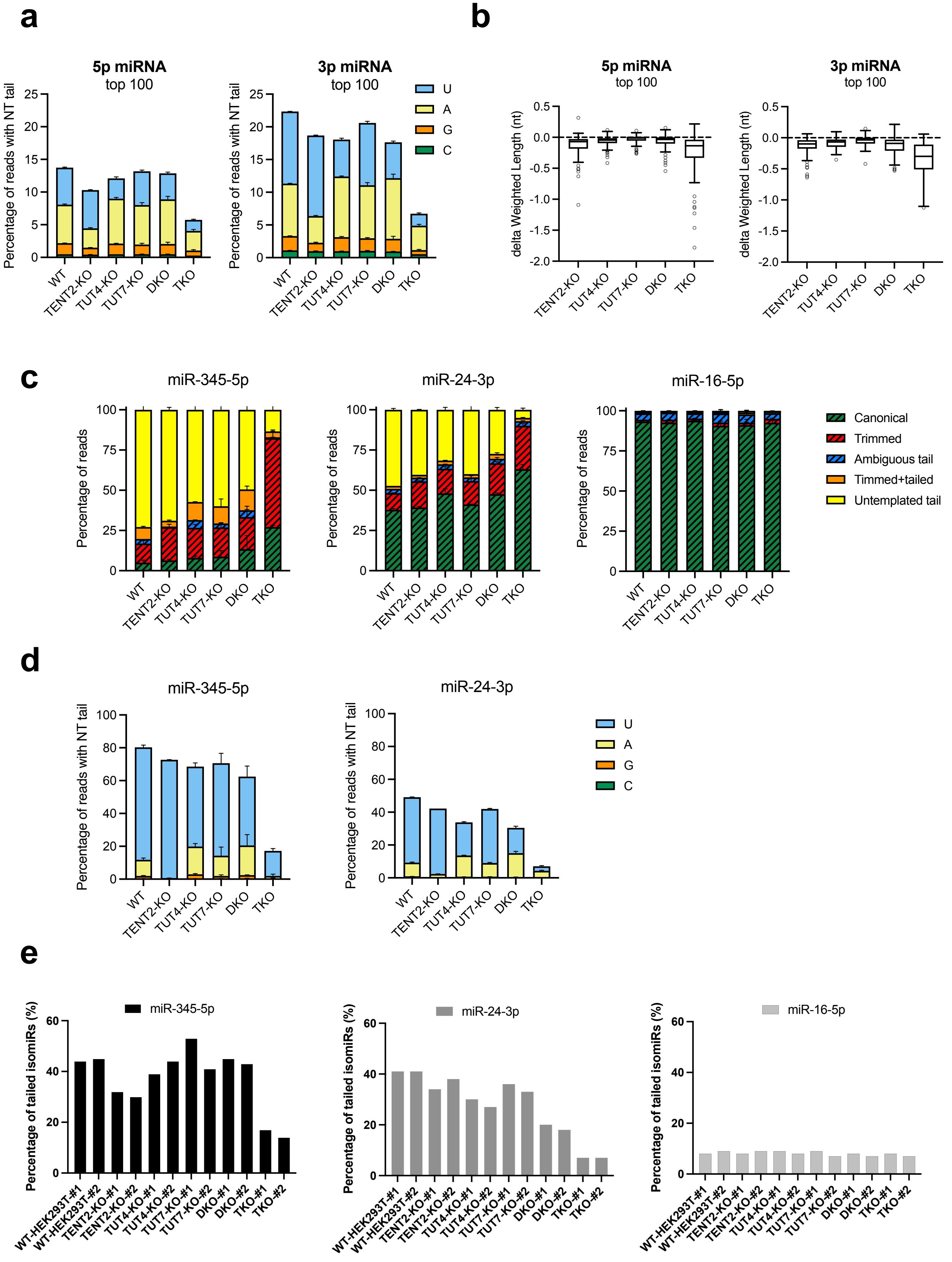
a
5p miRNA
top 100
Percentage of reads with NT tail
WT
TENT2-KO
TUT4-KO
TUT7-KO
DKO
TKO
3p miRNA
top 100
U
A
G
C
b
5p miRNA
top 100
delta Weighted Length (nt)
3p miRNA
top 100
c
miR-345-5p
miR-24-3p
miR-16-5p
Percentage of reads
Canonical
Trimmed
Ambiguous tail
Timmed+tailed
Untemplated tail
d
miR-345-5p
miR-24-3p
Percentage of reads with NT tail
e
miR-345-5p
miR-24-3p
miR-16-5p
Percentage of tailed isomiRs (%)
WT-HEK293T-#1
WT-HEK293T-#2
TENT2-KO-#1
TENT2-KO-#2
TUT4-KO-#1
TUT4-KO-#2
TUT7-KO-#1
TUT7-KO-#2
DKO-#1
DKO-#2
TKO-#1
TKO-#2

Supplementary Fig. 2 Coordination of TENT2, TUT4 and TUT7 in miRNA 3' tailing.

a Nucleotide identity on NT tails in the different knockout clones on the top 100 most abundant 5p and 3p miRNAs. Column height represents the percentage of NT tail while colored areas represent the corresponding fraction based on nucleotide identity (Average ± standard error, N=100 5p miRNAs and N=100 3p miRNAs). **b** Box-plot of the average length change (delta) between the different knockouts and WT cells. Average lengths were weighted based on relative isomiR abundances within each miRNA (center median, whiskers based on Tukey test, N=100 5p miRNAs and N=100 3p miRNAs). **c, d** Analysis of the 3' end composition (**c**) and nucleotide identity (**d**) based on miRNA-seq data from WT and the different knockout cells (Average ± standard error, N=2 biologically independent samples or clones). **e** The percentage of the tailed isomiRs was calculated based on the ratio of the tailed isomiR bands with all bands in the NB data shown in Fig. 2d.

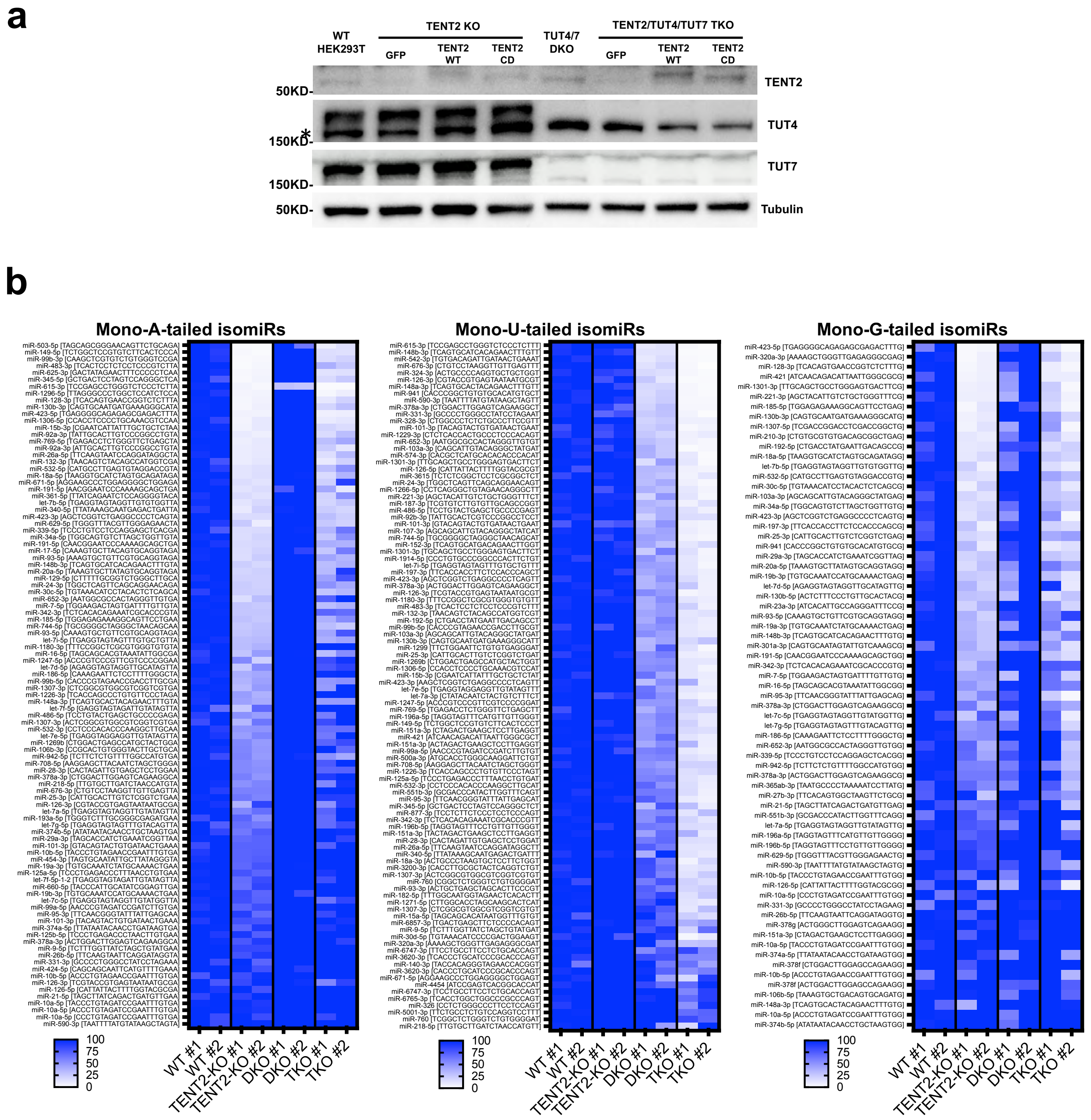
a
WT HEK293T
TENT2 KO
GFP
TENT2 WT
TENT2 CD
TUT4/7 DKO
TENT2/TUT4/TUT7 TKO
GFP
TENT2 WT
TENT2 CD
50KD-
TENT2
150KD-
TUT4
150KD-
TUT7
50KD-
Tubulin
b
Mono-A-tailed isomiRs
Mono-U-tailed isomiRs
Mono-G-tailed isomiRs
100
75
50
25
0
WT #1
WT #2
TENT2-KO #1
TENT2-KO #2
DKO #1
DKO #2
TKO #1
TKO #2

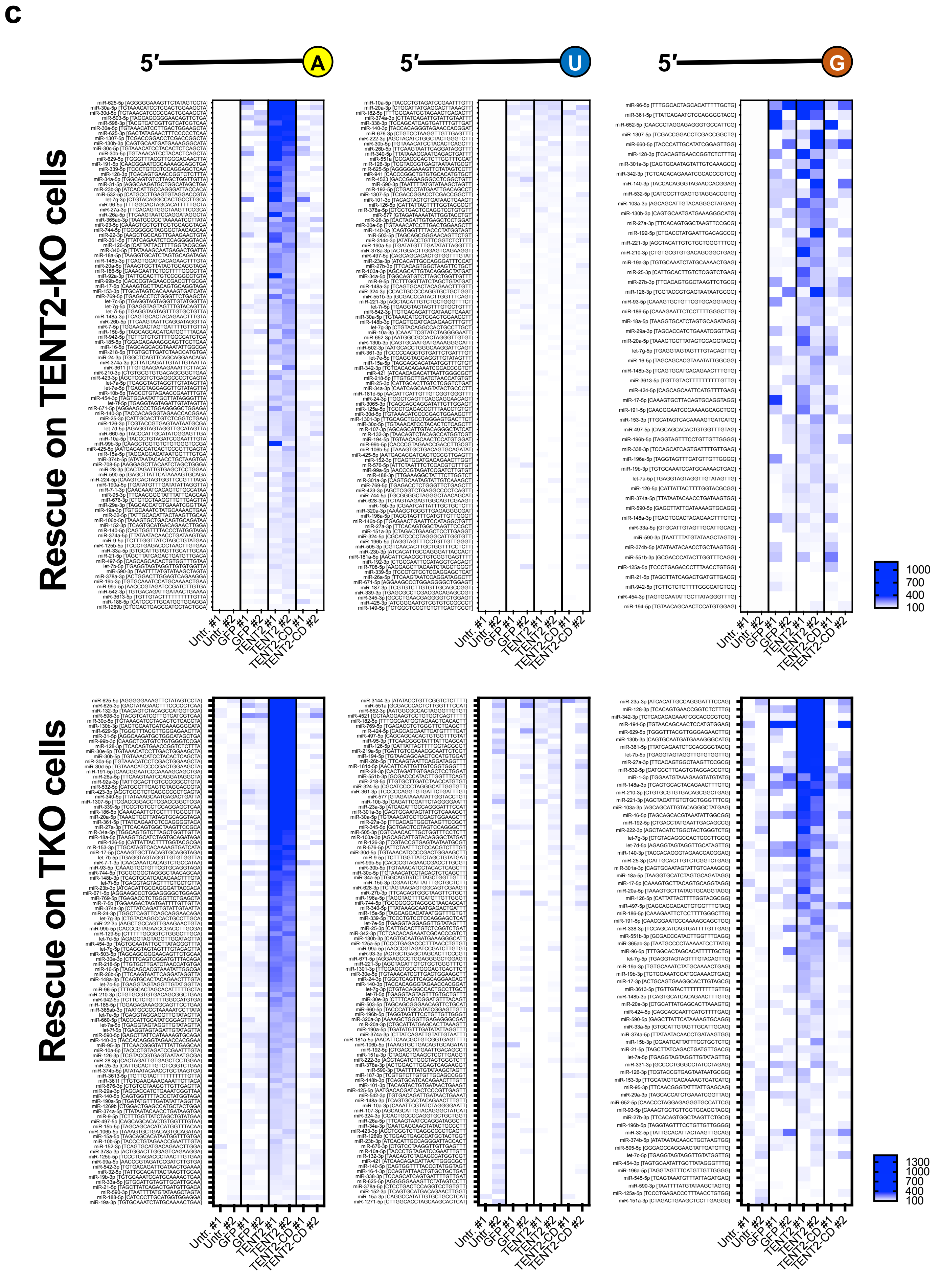

c
5′ A
5′ U
5′ G
Rescue on TENT2-KO cells
Rescue on TKO cells
Untr. #1
Untr. #2
GFP #1
GFP #2
TENT2 #1
TENT2 #2
TENT2-CD #1
TENT2-CD #2
1000
700
400
100
1300
1000
700
400
100

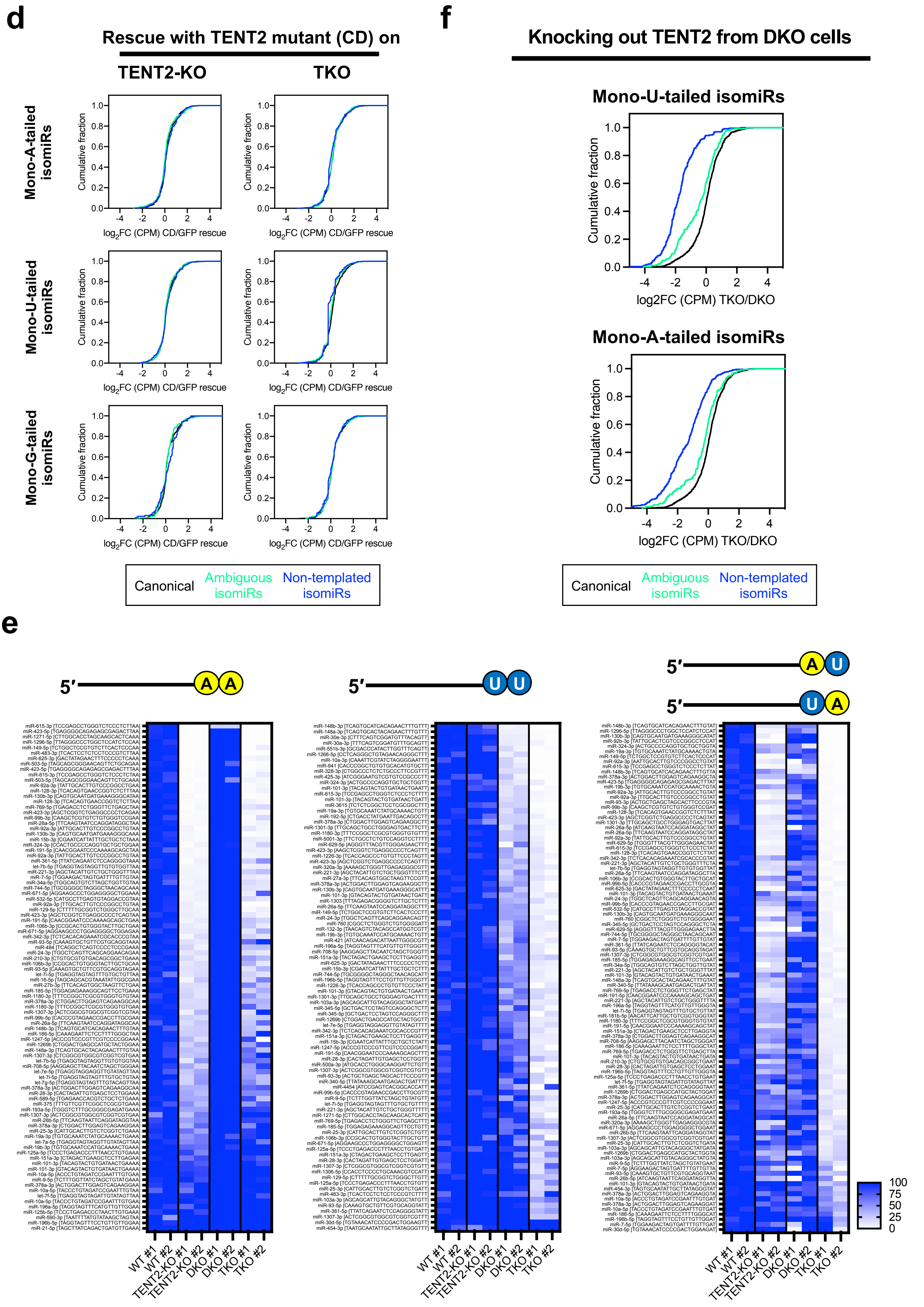
d
Rescue with TENT2 mutant (CD) on
TENT2-KO
TKO
Mono-A-tailed isomiRs
Mono-U-tailed isomiRs
Mono-G-tailed isomiRs
Cumulative fraction
log2FC (CPM) CD/GFP rescue
Canonical
Ambiguous isomiRs
Non-templated isomiRs
f
Knocking out TENT2 from DKO cells
Mono-U-tailed isomiRs
Mono-A-tailed isomiRs
Cumulative fraction
log2FC (CPM) TKO/DKO
Canonical
Ambiguous isomiRs
Non-templated isomiRs
e
5′
A A
U U
A U
U A
WT #1
WT #2
TENT2-KO #1
TENT2-KO #2
DKO #1
DKO #2
TKO #1
TKO #2
100
75
50
25
0

g

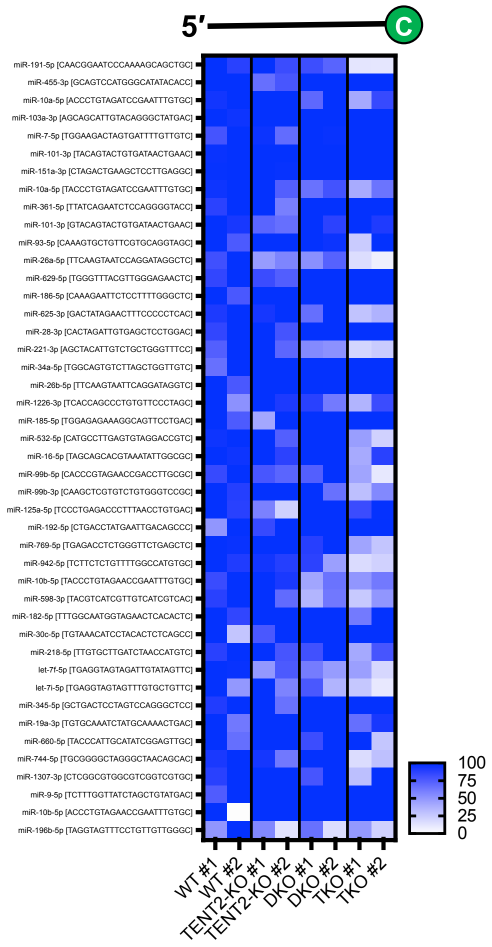

Supplementary Fig. 3 TENT2 contributes to adenylation, uridylation and guanylation of miRNAs.

a Western blot validating TENT2-WT and TENT2-CD (catalytic dead) rescues in TENT2-KO and DKO cells. Asterisk indicates non-specific bands. Of note, ectopically expressed TENT2-WT was FLAG-tagged at the N terminal and run slower than the endogenous TENT2. **b** Heatmaps of the relative abundance of mono-tailed isomiRs (sequences are indicated in square brackets) between WT and knockout cells. Colorimetric scale ranges in values between 0 and ≥100. **c** Heatmaps of the relative abundance of mono-tailed isomiRs between knockout cells and rescues with GFP, TENT2-WT and TENT2-CD. Colorimetric scale ranges in values between 100 and the maximum observed values. **d** Cumulative curves of all mono-tailed isomiRs upon rescue with TENT2-CD or GFP in TENT2-KO and TKO cells. Colored lines indicate canonical miRNAs (black), templated/ambiguous (green) and NT (blue) isomiRs. **e** Heatmaps of the relative abundance of di-tailed ["AA", "UU" or "AU/UA"] isomiRs between WT and knockout cells. Colorimetric scale ranges in values between 0 and ≥100. **f** Cumulative curves comparing the effect of TENT2 knockout on the background of DKO on total miRNA. **g** Heatmaps of the relative abundance of "C" mono-tailed isomiRs between WT and knockout cells. Colorimetric scale ranges in values between 0 and ≥100.

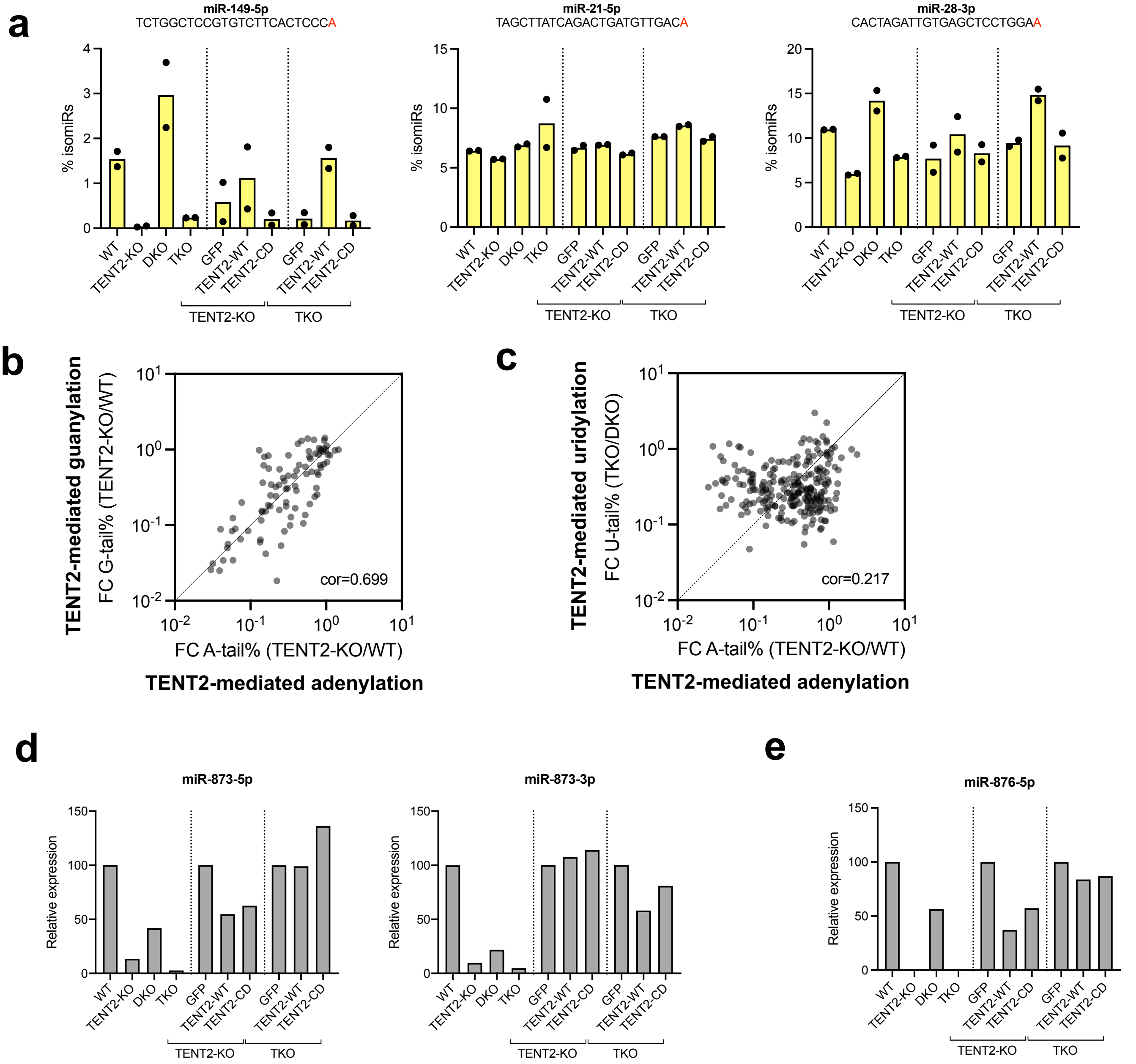

Supplementary Fig. 4 TENT2 selectively modifies mature miRNAs.

a Percentage of adenylated isomiRs across different knockouts and rescues (N=2 biologically independent samples or clones). Sequences indicate templated nucleotides (black) and NT adenylation (red). **b** Scatter plot of fold-change in the percentage of NT A-tail and G-tail (matched isomiRs) between WT and TENT2-KO. **c** Scatter plot of fold-change in the percentage of NT A-tail and U-tail (matched isomiRs) between different genetic backgrounds. Correlation (cor) was calculated using the Pearson coefficient. **d, e** Overall miRNA abundance (counts per million, CPM) for miR-873-5p, miR-873-3p (**d**) and miR-876-5p (**e**) across different knockouts and rescues.

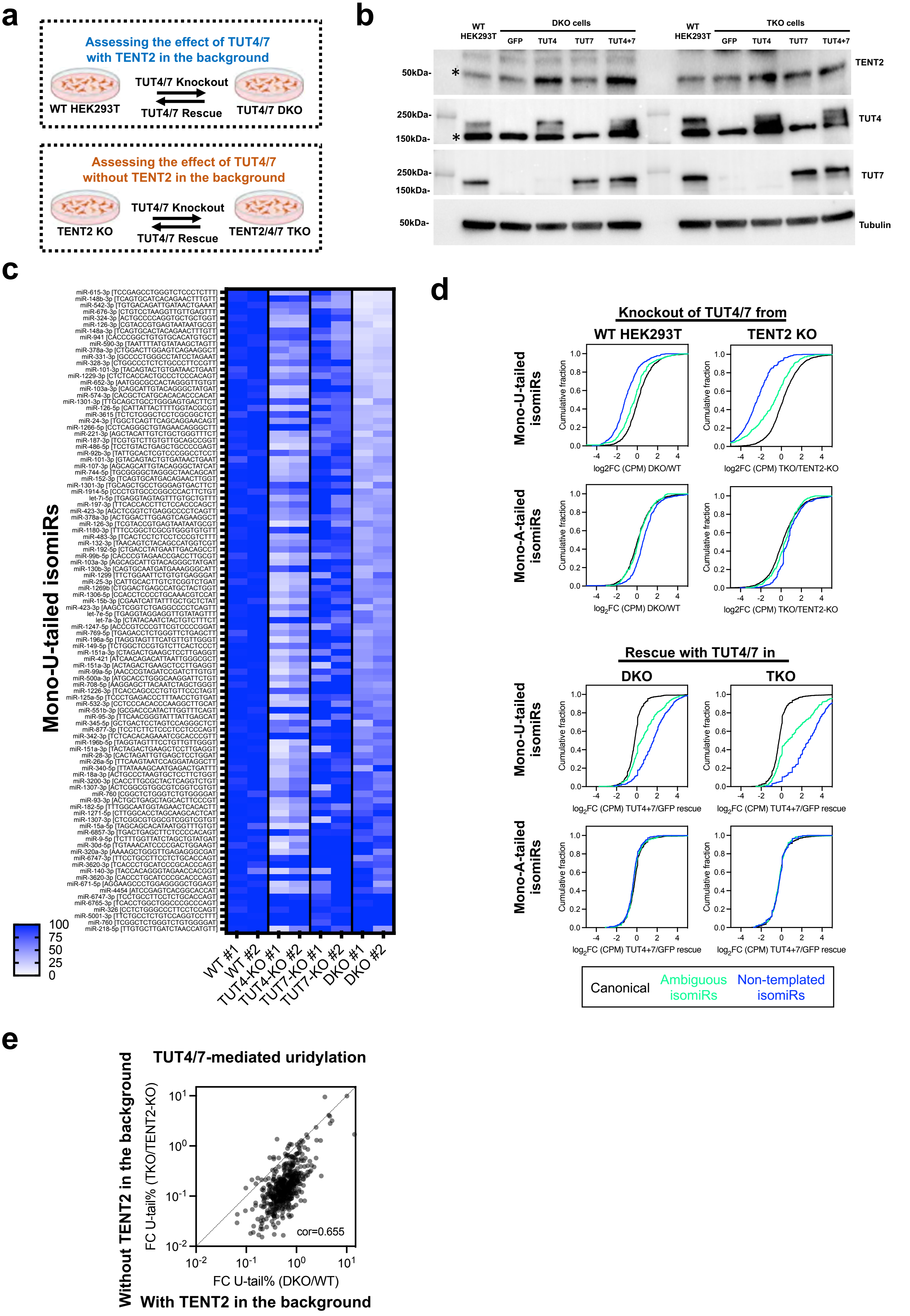

a
Assessing the effect of TUT4/7 with TENT2 in the background
WT HEK293T
TUT4/7 Knockout
TUT4/7 Rescue
TUT4/7 DKO
Assessing the effect of TUT4/7 without TENT2 in the background
TENT2 KO
TUT4/7 Knockout
TUT4/7 Rescue
TENT2/4/7 TKO
b
WT HEK293T
DKO cells
GFP
TUT4
TUT7
TUT4+7
WT HEK293T
TKO cells
GFP
TUT4
TUT7
TUT4+7
50kDa-
TENT2
250kDa-
150kDa-
TUT4
250kDa-
150kDa-
TUT7
50kDa-
Tubulin
c
Mono-U-tailed isomiRs
100
75
50
25
0
WT #1
WT #2
TUT4-KO #1
TUT4-KO #2
TUT7-KO #1
TUT7-KO #2
DKO #1
DKO #2
d
Knockout of TUT4/7 from
WT HEK293T
TENT2 KO
Mono-U-tailed isomiRs
Mono-A-tailed isomiRs
Cumulative fraction
log2FC (CPM) DKO/WT
log2FC (CPM) TKO/TENT2-KO
Rescue with TUT4/7 in
DKO
TKO
log2FC (CPM) TUT4+7/GFP rescue
Canonical
Ambiguous isomiRs
Non-templated isomiRs
e
TUT4/7-mediated uridylation
Without TENT2 in the background
FC U-tail% (TKO/TENT2-KO)
FC U-tail% (DKO/WT)
With TENT2 in the background
cor=0.655

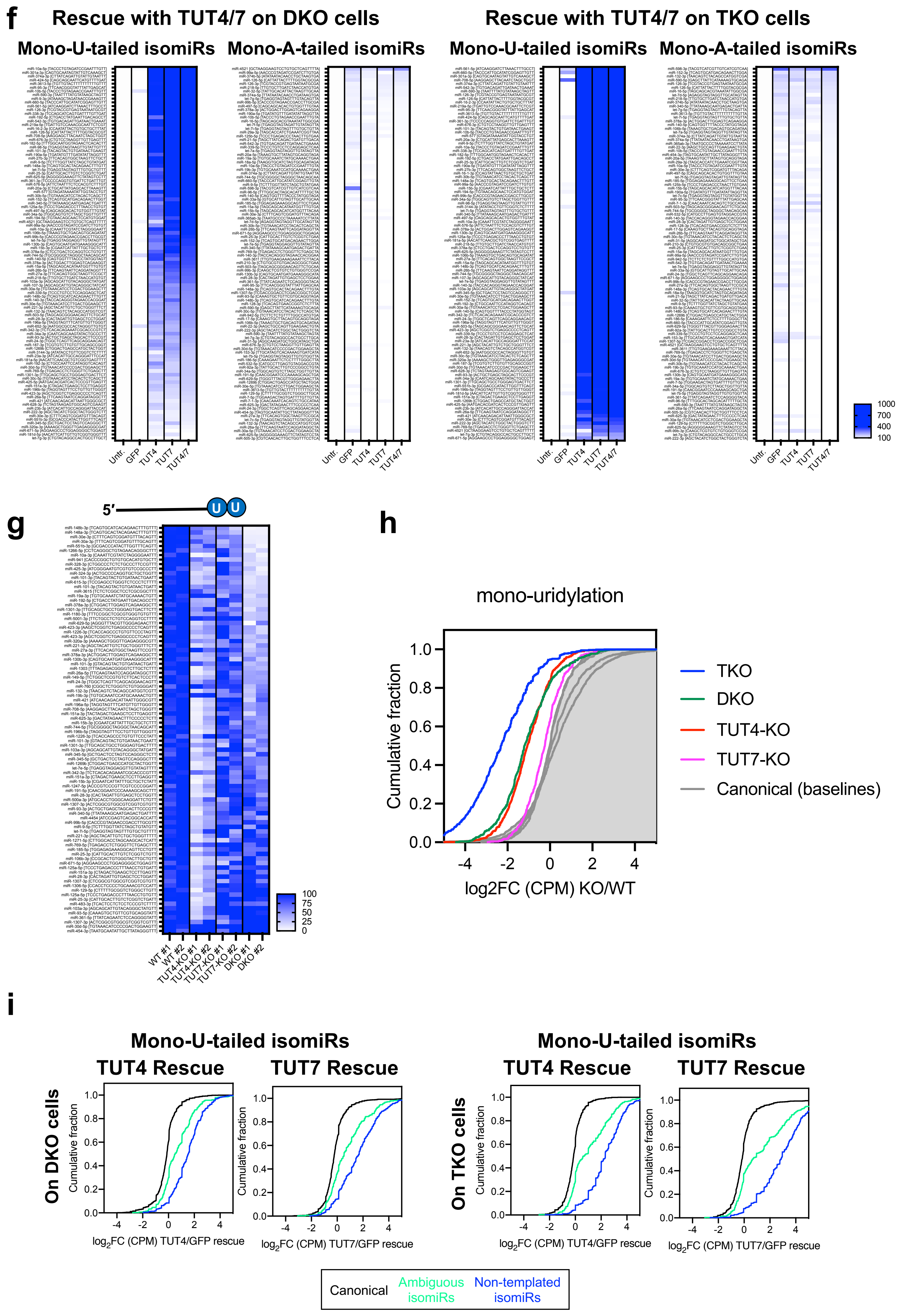
f
Rescue with TUT4/7 on DKO cells
Rescue with TUT4/7 on TKO cells
Mono-U-tailed isomiRs
Mono-A-tailed isomiRs
Mono-U-tailed isomiRs
Mono-A-tailed isomiRs
Untr.
GFP
TUT4
TUT7
TUT4/7
1000
700
400
100
g
5′
U
U
100
75
50
25
0
WT #1
WT #2
TUT4-KO #1
TUT4-KO #2
TUT7-KO #1
TUT7-KO #2
DKO #1
DKO #2
h
mono-uridylation
Cumulative fraction
log2FC (CPM) KO/WT
TKO
DKO
TUT4-KO
TUT7-KO
Canonical (baselines)
i
Mono-U-tailed isomiRs
TUT4 Rescue
TUT7 Rescue
On DKO cells
On TKO cells
Cumulative fraction
log2FC (CPM) TUT4/GFP rescue
log2FC (CPM) TUT7/GFP rescue
Canonical
Ambiguous isomiRs
Non-templated isomiRs

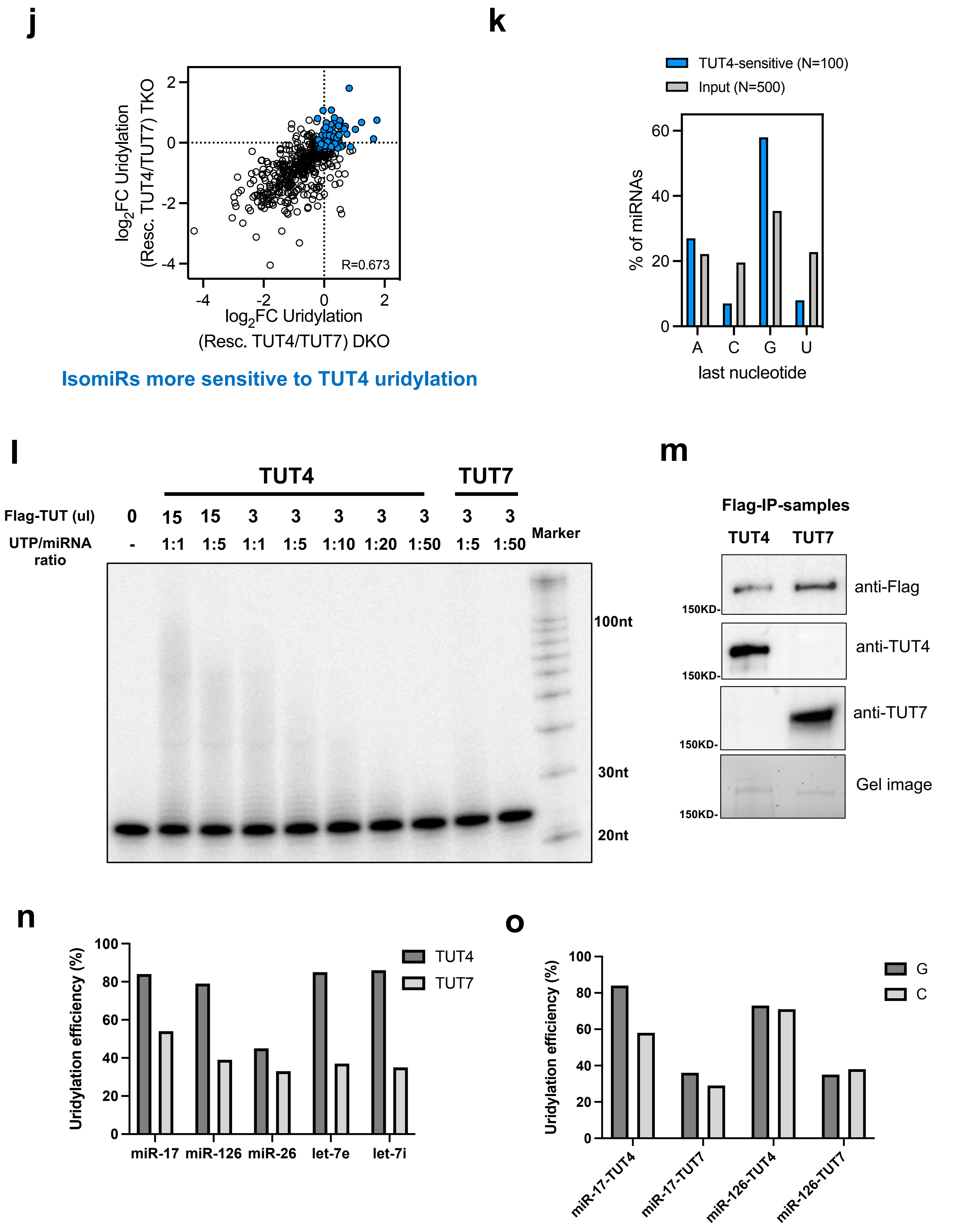
j
log2FC Uridylation (Resc. TUT4/TUT7) TKO
log2FC Uridylation (Resc. TUT4/TUT7) DKO
R=0.673
IsomiRs more sensitive to TUT4 uridylation
k
TUT4-sensitive (N=100)
Input (N=500)
% of miRNAs
last nucleotide
A
C
G
U
l
TUT4
TUT7
Flag-TUT (ul)
UTP/miRNA ratio
0
15
15
3
3
3
3
3
3
3
-
1:1
1:5
1:1
1:5
1:10
1:20
1:50
1:5
1:50
Marker
100nt
30nt
20nt
m
Flag-IP-samples
TUT4
TUT7
anti-Flag
anti-TUT4
anti-TUT7
Gel image
150KD-
n
Uridylation efficiency (%)
TUT4
TUT7
miR-17
miR-126
miR-26
let-7e
let-7i
o
Uridylation efficiency (%)
G
C
miR-17-TUT4
miR-17-TUT7
miR-126-TUT4
miR-126-TUT7

Supplementary Fig. 5 TUT4 but not TUT7 selectively uridylates most mature miRNAs.

a Scheme of the experimental strategy. Created with BioRender.com **b** Western blot validation of TUT4 and TUT7 single and double rescues in DKO and TKO cells. Asterisk indicates non-specific bands. **c** Heatmap of the relative abundance of mono-uridylated isomiRs between WT and knockout cells. Colorimetric scale ranges in values between 0 and ≥100. **d** Cumulative curves of all mono-uridylated (upper panel) and mono-adenylated (lower panel) isomiRs upon knockout or rescue of TUT4 and TUT7 in different cellular knockout backgrounds. Colored lines indicate canonical miRNAs (black), templated/ambiguous (green) and NT (blue) isomiRs. **e** Scatter plot of fold-changes in the percentage of NT U-tail upon the knockout of TUT4/7 in different genetic backgrounds. **f** Heatmaps of the relative abundance of mono-tailed isomiRs between knockout cells and rescues with GFP, TUT4, TUT7 and TUT4/7. Colorimetric scale ranges in values between 100 and the maximum observed values. **g** Heatmaps of the relative abundance of di-uridylated "UU" isomiRs between WT and knockout cells. Colorimetric scale ranges in values between 0 and ≥100. **h** Cumulative curves of all mono-uridylated isomiRs in different knockout cells (colored) compared to the corresponding baselines, canonical isoforms (gray). **i** Cumulative curves of all mono-uridylated isomiRs upon rescue with TUT4 and TUT7 in different cellular knockout backgrounds. Colored lines indicate canonical miRNAs (black), templated/ambiguous (green) and NT (blue) isomiRs. **j** Scatter plot of fold-changes in the percentage of NT U-tail upon the rescue of TUT4 or TUT7 in different genetic backgrounds. Dots highlighted in blue indicate a subset of isomiRs more sensitive to TUT4 uridylation. **k** Analysis of TUT4 tailing preference based on the last templated nucleotide preceding the uridylation. The TUT4-sensitive group (N=100) was defined based on the TUT4 rescue response described in Supplementary Fig. 5j. **l** Optimization of the in vitro uridylation assay of synthetic miRNAs. Conditions optimized included enzyme concentration (Flag-TUT) and molar ratios of UTP and miRNA. **m** Western blot analysis to validate immuno-purified TUT4 and TUT7 proteins. **n** and **o** For uridylation assays shown in Fig. 5f and 5g, total substrate amounts were determined by quantifying the bands in the mock-treated lanes. Un-uridylated bands were quantified for each treatment and the uridylation efficiency was calculated accordingly.

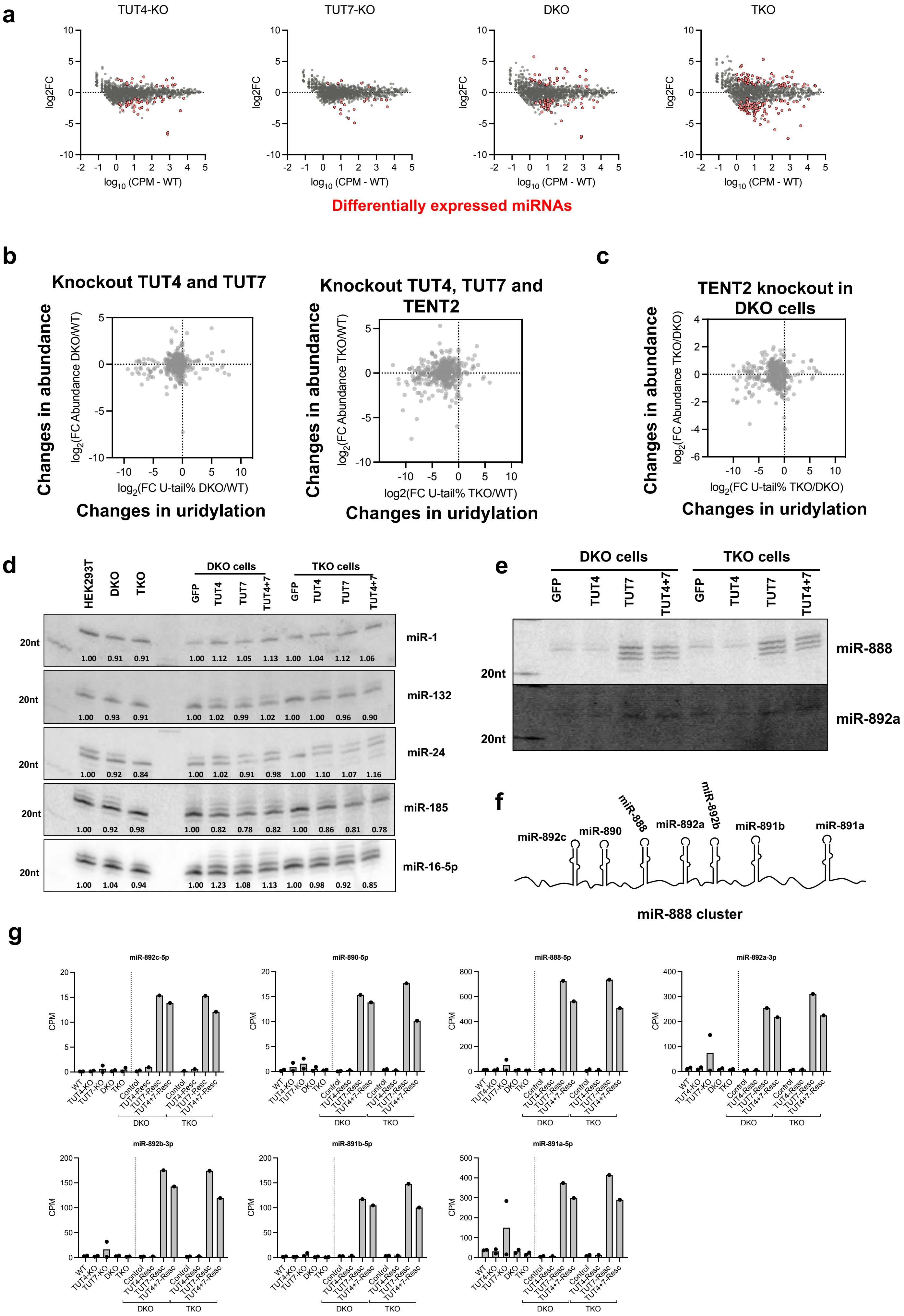
a
TUT4-KO
TUT7-KO
DKO
TKO
log2FC
log10 (CPM - WT)
Differentially expressed miRNAs
b
Knockout TUT4 and TUT7
Changes in abundance
log2(FC Abundance DKO/WT)
log2(FC U-tail% DKO/WT)
Changes in uridylation
Knockout TUT4, TUT7 and TENT2
log2(FC Abundance TKO/WT)
log2(FC U-tail% TKO/WT)
c
TENT2 knockout in DKO cells
log2(FC Abundance TKO/DKO)
log2(FC U-tail% TKO/DKO)
d
HEK293T
DKO
TKO
DKO cells
TKO cells
GFP
TUT4
TUT7
TUT4+7
20nt
miR-1
1.00 0.91 0.91 1.00 1.12 1.05 1.13 1.00 1.04 1.12 1.06
miR-132
1.00 0.93 0.91 1.00 1.02 0.99 1.02 1.00 1.00 0.96 0.90
miR-24
1.00 0.92 0.84 1.00 1.02 0.91 0.98 1.00 1.10 1.07 1.16
miR-185
1.00 0.92 0.98 1.00 0.82 0.78 0.82 1.00 0.86 0.81 0.78
miR-16-5p
1.00 1.04 0.94 1.00 1.23 1.08 1.13 1.00 0.98 0.92 0.85
e
DKO cells
TKO cells
GFP
TUT4
TUT7
TUT4+7
20nt
miR-888
miR-892a
f
miR-892c
miR-890
miR-888
miR-892a
miR-892b
miR-891b
miR-891a
miR-888 cluster
g
miR-892c-5p
miR-890-5p
miR-888-5p
miR-892a-3p
miR-892b-3p
miR-891b-5p
miR-891a-5p
CPM
WT
TUT4-KO
TUT7-KO
DKO
TKO
Control
TUT4-Resc
TUT7-Resc
TUT4+7-Resc
DKO
TKO

Supplementary Fig. 6 TUT4/7 regulate the abundance of a set of miRNAs via distinct mechanisms.

a Scatter plots of differentially expressed miRNA in different knockouts compared to wild-type cells (red dots). **b, c** Scatter plot of fold-changes in the percentage of NT U-tail against fold-changes in miRNA abundance in different genetic backgrounds. **d** Northern blot validation of the expression changes of miR-1, -132, -24, -185, and -16 in WT, and DKO or TKO cells with or without rescue. **e** Northern blot validation of the expression changes of miR-888 and miR-892a in DKO and TKO cells with or without rescue. **f** Scheme of the miR-890 cluster. **g** Quantification of changes in expression (CPM, counts per million) of members of the miR-890 cluster in different TUT knockouts (N=2 biologically independent samples or clones) and rescues (N=1 experiment).

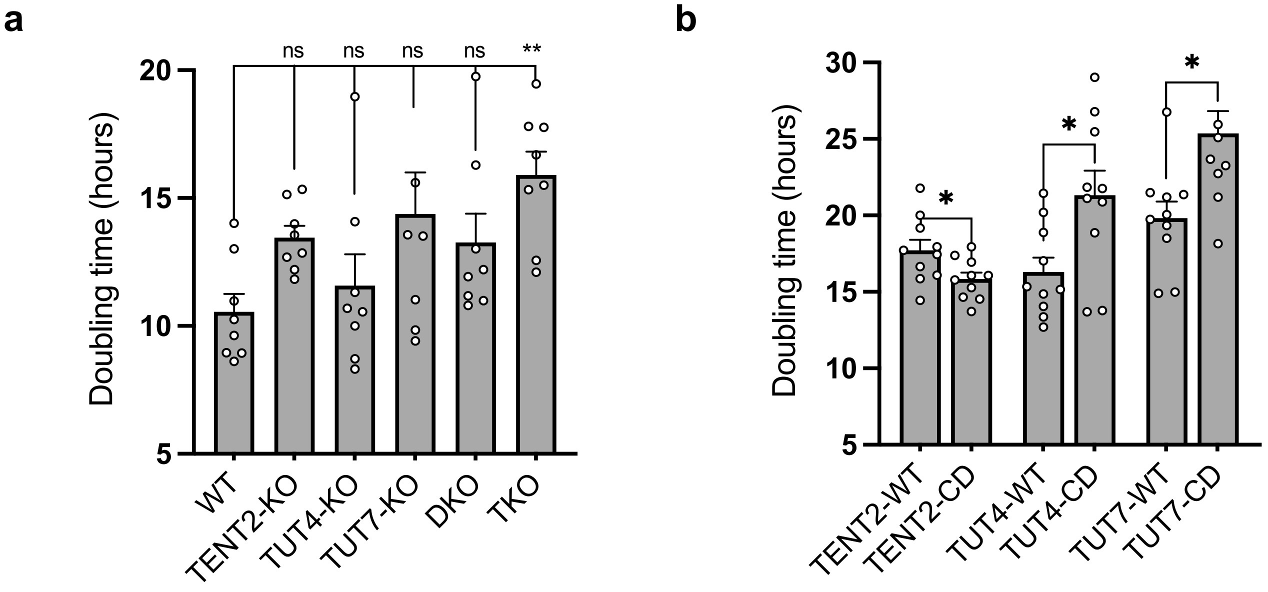

Supplementary Fig. 7 TUT4 and TUT7 promote growth of the HEK293T cells.
a Cell growth doubling time in different TENT knockout cells compared to wild-type cells. Each group contains biological replicates of two independent clones (Average ± standard error, N=8). Data were analyzed using the Friedman test and Dunn's multiple comparison test (two-sided). **p–value smaller than 0.01 (exact p-value: 0.0067). **b** Cell growth doubling time of TKO cells rescued with different TENTs and corresponding catalytic-dead mutants. Each group contained biological replicates of two independent clones (Average ± standard error, N=10). Data was analyzed using the Mann-Whitney's U test (two-sided). * equal p–value smaller than 0.05 (exact p-values: 0.0355, 0.0288 and 0.0115, respectively).

miRNA positive regulation by TUT4/7	
**miRNA name**	**Expression responsive to rescue with**
hsa-miR-1-3p	TUT4, TUT7, TUT4+7
hsa-miR-132-3p	TUT4, TUT7, TUT4+7
hsa-miR-1468-5p	TUT4, TUT7, TUT4+7
hsa-miR-185-5p	TUT4, TUT7, TUT4+7
hsa-miR-203a-3p	TUT4, TUT7, TUT4+7
hsa-miR-24-3p	TUT4, TUT7, TUT4+7
hsa-miR-4454	TUT4, TUT7, TUT4+7
hsa-miR-615-3p	TUT4, TUT7, TUT4+7
hsa-miR-891b	TUT4, TUT7, TUT4+7
hsa-miR-124-3p	TUT7, TUT4+7
hsa-miR-124-5p	TUT7, TUT4+7
hsa-miR-1257	TUT7, TUT4+7
hsa-miR-146a-5p	TUT7, TUT4+7
hsa-miR-199b-5p	TUT7, TUT4+7
hsa-miR-301b-3p	TUT7, TUT4+7
hsa-miR-34b-5p	TUT7, TUT4+7
hsa-miR-34c-5p	TUT7, TUT4+7
hsa-miR-548d-3p	TUT7, TUT4+7
hsa-miR-651-5p	TUT7, TUT4+7
hsa-miR-653-3p	TUT7, TUT4+7
hsa-miR-890	TUT7, TUT4+7
hsa-miR-892b	TUT7, TUT4+7
**miRNA negative regulation by TUT4/7**	
**miRNA name**	**Expression responsive to rescue with**
hsa-let-7b-5p	TUT4, TUT7, TUT4+7
hsa-let-7d-3p	TUT4, TUT7, TUT4+7
hsa-let-7d-5p	TUT4, TUT7, TUT4+7
hsa-let-7i-5p	TUT4, TUT7, TUT4+7
hsa-miR-1269b	TUT4, TUT7, TUT4+7
hsa-miR-141-5p	TUT4, TUT7, TUT4+7
hsa-miR-181b-5p	TUT4, TUT7, TUT4+7
hsa-miR-29b-2-5p	TUT4, TUT7, TUT4+7
hsa-miR-3187-3p	TUT4, TUT7, TUT4+7
hsa-miR-4470	TUT4, TUT7, TUT4+7
hsa-miR-4783-3p	TUT4, TUT7, TUT4+7
hsa-miR-671-5p	TUT4, TUT7, TUT4+7
hsa-miR-760	TUT4, TUT7, TUT4+7
hsa-miR-887-3p	TUT4, TUT7, TUT4+7
hsa-miR-92b-5p	TUT4, TUT7, TUT4+7
hsa-miR-1229-3p	TUT7, TUT4+7
hsa-miR-1284	TUT7, TUT4+7
hsa-miR-1301-3p	TUT7, TUT4+7

hsa-miR-138-5p	TUT7, TUT4+7
hsa-miR-181d-5p	TUT7, TUT4+7
hsa-miR-2278	TUT7, TUT4+7
hsa-miR-29b-1-5p	TUT7, TUT4+7
hsa-miR-3176	TUT7, TUT4+7
hsa-miR-4326	TUT7, TUT4+7
hsa-miR-5682	TUT7, TUT4+7
hsa-miR-628-3p	TUT7, TUT4+7
hsa-miR-628-5p	TUT7, TUT4+7
hsa-miR-3928-3p	TUT4, TUT4+7

Supplementary Table S1 miRNAs regulated by TUT4/7

	Northern blotting probes
hsa-miR-345-5p probe	GAGCCCTGGACTAGGAGTCAGC
hsa-miR-24-3p probe	GCTGTTCCTGCTGAACTGAGCCA
hsa-miR-16-5p probe	GCGCCAATATTTACGTGCTGCTA
hsa-Let-7g-5p probe	GAACTGTACAAACTACTACCTCA
hsa-Let-7i-5p probe	GAACAGCACAAACTACTACCTCA
hsa-miR-222-3p probe	GACCCAGTAGCCAGATGTAGCT
hsa-miR-221-3p probe	GAAACCCAGCAGACAATGTAGCT
hsa-miR-181b-5p probe	GACCCACCGACAGCAATGAATGTT
hsa-miR-181a-5p probe	GACTCACCGACAGCGTTGAATGTT
hsa-miR-888-5p probe	GTGACTGACAGCTTTTTGAGTA
hsa-miR-892a-3p probe	GCTACGCAGAAAGGACACAGTG
	**Infusion cloning primers**
TENT2-Infusion-F	CCCGATTACGCTAGCTTCCCAAACTCAATTTTGGGTCGCC
TENT2-Infusion-R	GGATCCACTGAATTCTTATCTTTTCAGGACAGCAGCTCTT
TUT4-Infusion-F	CCCGATTACGCTAGCGAAGAGTCTAAAACCTTAAAAAGTG
TUT4-Infusion-R	GGATCCACTGAATTCTTACTCCGACACGTTTCCTCTTGGT
TUT7-Infusion-F	CCCGATTACGCTAGCGGAGATACAGCAAAACCTTATTTCG
TUT7-Infusion-R	GGATCCACTGAATTCTCATGATTCCTGCTGGGTCCTCTTC
	**catalytic dead mutagenesis primers**
TENT2-mut-F	TGCTTTATGCCTAGTTGTTAAGGAAGAAC
TENT2-mut-R	CCAGCACTGCTCCGGGTACCAAA
TUT4-mut-F	GGCTATTTGTATGACCCTGGAAG
TUT4-mut-R	AGAGCACTATCACGAAATCCAAATC
TUT7-mut-F	TGCCGTCTGTATGACAATTAATGG
TUT7-mut-R	AGGGCACTCTGTTTGAACCCAAATC
	**sgRNA sequences**
TENT2-gRNA-1	ATTGTGCATCTATAAGCTGC
TENT2-gRNA-2	AAGTGCCAATTGTGAAGTTC
TENT2-gRNA-3	TGAATAAACAGTAGGTGACA
TENT2-gRNA-4	TAAATCACCATCACTGCTCC
TUT4-gRNA-1	TGCAGATTCATCTATCACAG
TUT4gRNA-2	CTGCATTGACACCTGAGCAG
TUT4-gRNA-3	TGGATTTCGTGATAGTGATC
TUT4-gRNA-4	GGCTCTTCTAAGAATGGATT
TUT7-gRNA-1	AGTACAGACTCATCGATCAC
TUT7-gRNA-2	TTTGAAACCCAATCTGCTAC
TUT7-gRNA-3	TGAGTCTGTACTTTCAACGA
TUT7-gRNA-4	GGGTCATCCTGTAGCAGATT
	**synthetic miRNA sequences**
hsa-miR-17-5p	rCrArArArGrUrGrCrUrUrArCrArGrUrGrCrArGrGrUrArG
hsa-miR-126-5p	rCrArUrUrArUrUrArCrUrUrUrUrGrGrUrArCrGrCrG
hsa-miR-26a-5p	rUrUrCrArArGrUrArArUrCrCrArGrGrArUrArGrGrCrU
hsa-let-7e-5p	rUrGrArGrGrUrArGrGrArGrGrUrUrGrUrArUrArGrUrU
hsa-let-7i-5p	rUrGrArGrGrUrArGrUrArGrUrUrUrGrUrGrCrUrGrUrU
hsa-miR-17-5p-C	rCrArArArGrUrGrCrUrUrArCrArGrUrGrCrArGrGrUrArC
hsa-miR-126-5p-C	rCrArUrUrArUrUrArCrUrUrUrUrGrGrUrArCrGrCrC

Supplementary Table S2 Sequences of oligos used in this study
