# Supplementary material for: TENT2, TUT4, and TUT7 selectively regulate miRNA sequence and abundance
Source: Nat Commun. 2022 Sep 7;13:5260. doi: 10.1038/s41467-022-32969-8 (PMC9452540; doi:10.1038/s41467-022-32969-8)
Supplement: Supplementary file 3 — Description of Additional Supplementary Files [file 41467_2022_32969_MOESM3_ESM.pdf]

Description of Additional Supplementary Files

File Name: Supplementary Data 1
Description: Summary of miRNA expressions and isomiR composition
