# Supplementary material for: TENT2, TUT4, and TUT7 selectively regulate miRNA sequence and abundance
Source: Nat Commun. 2022 Sep 7;13:5260. doi: 10.1038/s41467-022-32969-8 (PMC9452540; doi:10.1038/s41467-022-32969-8)
Supplement: Supplementary file 5 — Reporting Summary [file 41467_2022_32969_MOESM5_ESM.pdf]

nature portfolio

Corresponding author(s): Shuo Gu

Last updated by author(s): Aug 18, 2022

Reporting Summary

Nature Portfolio wishes to improve the reproducibility of the work that we publish. This form provides structure for consistency and transparency in reporting. For further information on Nature Portfolio policies, see our Editorial Policies and the Editorial Policy Checklist.

Statistics

For all statistical analyses, confirm that the following items are present in the figure legend, table legend, main text, or Methods section.

n/a	Confirmed	
☐	☒	The exact sample size (*n*) for each experimental group/condition, given as a discrete number and unit of measurement
☐	☒	A statement on whether measurements were taken from distinct samples or whether the same sample was measured repeatedly
☐	☒	The statistical test(s) used AND whether they are one- or two-sided<br>*Only common tests should be described solely by name; describe more complex techniques in the Methods section.*
☒	☐	A description of all covariates tested
☒	☐	A description of any assumptions or corrections, such as tests of normality and adjustment for multiple comparisons
☐	☒	A full description of the statistical parameters including central tendency (e.g. means) or other basic estimates (e.g. regression coefficient) AND variation (e.g. standard deviation) or associated estimates of uncertainty (e.g. confidence intervals)
☐	☒	For null hypothesis testing, the test statistic (e.g. *F*, *t*, *r*) with confidence intervals, effect sizes, degrees of freedom and *P* value noted<br>*Give P values as exact values whenever suitable.*
☒	☐	For Bayesian analysis, information on the choice of priors and Markov chain Monte Carlo settings
☒	☐	For hierarchical and complex designs, identification of the appropriate level for tests and full reporting of outcomes
☐	☒	Estimates of effect sizes (e.g. Cohen's *d*, Pearson's *r*), indicating how they were calculated

Our web collection on statistics for biologists contains articles on many of the points above.

Software and code

Policy information about availability of computer code

Data collection	miRNA-seq (Illumina Miseq/Nextseq)
Data analysis	Customized scripts (https://github.com/Gu-Lab-RBL-NCI/TUT-tailing/tree/main/processing%20miRNA), QuagmiR (Bioinformatics, 2018), Rstudio 2022.07.1 Build 554, Prism v.8, ImageJ 1.52a

For manuscripts utilizing custom algorithms or software that are central to the research but not yet described in published literature, software must be made available to editors and reviewers. We strongly encourage code deposition in a community repository (e.g. GitHub). See the Nature Portfolio guidelines for submitting code & software for further information.

Data

Policy information about availability of data

All manuscripts must include a data availability statement. This statement should provide the following information, where applicable:

- Accession codes, unique identifiers, or web links for publicly available datasets
- A description of any restrictions on data availability
- For clinical datasets or third party data, please ensure that the statement adheres to our policy

All the figures are included in this article (and its supplementary information files). The NGS datasets generated during and/or analyzed during the current study are available in the GEO (GSE183384), (GSE184550), and (GSE203472). The source data for all the plots and the raw data for all Western and Northern blots in all figures and supplementary figures are provided as a Source Data file, which is available at Mendeley (doi: 10.17632/5hb33bwd9m.2).

Field-specific reporting

Please select the one below that is the best fit for your research. If you are not sure, read the appropriate sections before making your selection.

☒ Life sciences ☐ Behavioural & social sciences ☐ Ecological, evolutionary & environmental sciences

For a reference copy of the document with all sections, see nature.com/documents/nr-reporting-summary-flat.pdf

Life sciences study design

All studies must disclose on these points even when the disclosure is negative.

Sample size	No sample size calculation was done. Sample sizes were selected based on the magnitude of the effects observed and previous literature.
Data exclusions	No data were excluded.
Replication	Experiments were replicated successful, and the number of replication were documented in the manuscript. Independent colonies of knock-outs were used to minimize off-target effects when possible, alternatively we had at least two biological replicates.
Randomization	Randomization is not relevant to this study. Isogenic cell lines were treated with all treatments and controls in each case.
Blinding	Blinding is not relevant to this study. Isogenic cell lines were treated with all treatments and controls in each case.

Reporting for specific materials, systems and methods

We require information from authors about some types of materials, experimental systems and methods used in many studies. Here, indicate whether each material, system or method listed is relevant to your study. If you are not sure if a list item applies to your research, read the appropriate section before selecting a response.

Materials & experimental systems

n/a	Involved in the study	
☐	☒	Antibodies
☐	☒	Eukaryotic cell lines
☒	☐	Palaeontology and archaeology
☒	☐	Animals and other organisms
☒	☐	Human research participants
☒	☐	Clinical data
☒	☐	Dual use research of concern

Methods

n/a	Involved in the study	
☒	☐	ChIP-seq
☒	☐	Flow cytometry
☒	☐	MRI-based neuroimaging

Antibodies

Antibodies used	anti-Zcchc11 (Proteintech, #18980-1-AP, 1:500), anti-Zcchc6 (Proteintech, #25196-1-AP, 1:2000), rabbit anti-PAPD4 (TENT2, Abcam, #ab103884, 1:500), mouse anti-Flag (Sigma, #F1804, 1:3000), mouse anti-AGO1(2A7) (Wako, #015-22411, 5ug for IP), mouse anti-AGO2 (4G8) (Wako, #015-22031, 5ug for IP), and anti-tubulin (Sigma, #T9026, 1:5000).
Validation	anti-Zcchc11 (Proteintech, #18980-1-AP, 1:500) is validated by WB in MCF7 cells, HEK-293T cells, HeLa cells, human brain tissue and anti-Zcchc6 (Proteintech, #25196-1-AP, 1:2000) is validated by WB in PC-3 cells, HeLa cells, MCF-7 cells, mouse brain tissue as indicated in the Proteintech website. Rabbit anti-PAPD4 (TENT2, Abcam, #ab103884, 1:500) was validated by WB detecting TENT2 protein level in WT cells , KO cells and TENT2 over-expression samples (Fig. S3a). mouse anti-AGO1(2A7) (Wako, #015-22411, 5ug for IP) and mouse anti-AGO2 (4G8) (Wako, #015-22031, 5ug for IP) is suitable for IP for human protein as stated in the manufacturer's website. On Sigma's website, it's stated that Mouse anti-Flag (Sigma, #F1804, 1:3000) detects only the target protein band(s) on a Western blot from a mammalian crude cell lysate, and Monoclonal anti-tubulin (Sigma, #T9026, 1:5000) is immunospecific for tubulin as determined by indirect immunofluorescent staining and immunoblotting procedures. Good labeling may also be obtained with human, bovine, amphibian and mouse cells or tissues as well as with yeast or fungi.

Eukaryotic cell lines

Policy information about cell lines

Cell line source(s)	HEK293T is from ATCC. All the KO cell lines are established in the HEK293Tusing CRISPR-Cas9 system.
Authentication	HEK293T is authenticated by Short-tandem repeat profiling.

Mycoplasma contamination	HEK293T from ATCC is tested mycoplasma negtive.
Commonly misidentified lines (See ICLAC register)	none
